# Assessing alternative base substitutions at primer CpG sites to optimise unbiased PCR amplification of methylated sequences

**DOI:** 10.1186/s13148-017-0328-4

**Published:** 2017-04-04

**Authors:** Ida L. M. Candiloro, Thomas Mikeska, Alexander Dobrovic

**Affiliations:** 1 Peter MacCallum Cancer Centre, Melbourne, Victoria 3000 Australia; 2 Department of Pathology, The University of Melbourne, Parkville, Victoria 3010 Australia; 3 Translational Genomics and Epigenomics Laboratory, Olivia Newton-John Cancer Research Institute, Heidelberg, Victoria 3084 Australia; 4 La Trobe University, Bundoora, 3086 Victoria Australia

## Abstract

**Background:**

Determining the role of DNA methylation in various biological processes is dependent on the accurate representation of often highly complex patterns. Accurate representation is dependent on unbiased PCR amplification post bisulfite modification, regardless of methylation status of any given epiallele. This is highly dependent on primer design. Particular difficulties are raised by the analysis of CpG-rich regions, which are the usual regions of interest. Here, it is often difficult or impossible to avoid placing primers in CpG-free regions, particularly if one wants to target a specific part of a CpG-rich region. This can cause biased amplification of methylated sequences if the C is placed at those positions or to unmethylated sequences if a T is placed at those positions.

**Methods:**

We examined the effect of various base substitutions at the cytosine position of primer CpGs on the representational amplification of templates and also examined the role of the annealing temperature during PCR. These were evaluated using methylation-sensitive high-resolution melting and Pyrosequencing.

**Results:**

For a mixture of fully methylated and unmethylated templates, amplification using the C-, C/T (Y-) and inosine-containing primers was biased towards amplification of methylated DNA. The bias towards methylated sequences increased with annealing temperature. Amplification using primers with an A/C/G/T (N) degeneracy at the cytosine positions was not biased at the lowest temperature used but became increasingly biased towards methylated DNA with increased annealing temperature. Using primers matching neither C nor T was in the main unbiased but at the cost of poor PCR amplification efficiency. Primers with abasic sites were also unbiased but could only amplify DNA for one out of the two assays tested. However, with heterogeneous methylation, it appeared that both the primer type and stringency used have a minimal influence on PCR bias.

**Conclusions:**

This is the first comprehensive analysis of base substitutions at CpG sites in primers and their effect on PCR bias for the analysis of DNA methylation. Our findings are relevant to the appropriate design of a wide range of assays, including amplicon-based next-generation sequencing approaches that need to measure DNA methylation.

**Electronic supplementary material:**

The online version of this article (doi:10.1186/s13148-017-0328-4) contains supplementary material, which is available to authorized users.

## Background

Detection and quantification of DNA methylation usually relies on a PCR step after bisulfite modification. To achieve accurate representation, amplification should not be biased to either methylated or unmethylated sequences. A particular difficulty is raised by the analysis of CpG-rich regions typically studied in methylation analysis where primers containing CpG sequences favour the amplification of methylated sequences [1, 2]. Thus, for techniques that based on amplification of both methylated and unmethylated DNA such as methylation-sensitive high-resolution melting (MS-HRM), bisulfite pyrosequencing, Sanger sequencing and MassARRAY/EpiTYPER, PCR bias can lead to over- or underestimation of the true DNA methylation content [3].

Clark et al. [4] suggested that the omission of CpGs entirely from the primers should result in unbiased amplification. When methylation-independent primers are needed to amplify all epialleles regardless of methylation status, it is often difficult or impossible to avoid placing primers in CpG-free regions, particularly if one wants to target a specific part of the CpG-rich region in a short fragment. Thus, primers often need to be placed over CpG-containing sequences in the strand that is being amplified after bisulfite conversion. This can cause bias to the amplification of methylated sequences if the C is placed at the C of CpG sites, or to the amplification of unmethylated sequences if a T is placed at those sites.

In this report, we examined the effect of various substitutions at the cytosine position of the primer CpGs on the representational amplification of methylated templates. We chose regions from two genes that are often heterogeneously methylated in cancer and thus afford the opportunity to examine bias both in homogeneously and heterogeneously methylated contexts. We also examined the role of the annealing temperature during PCR for each of these substitutions.

## Methods

### DNA extraction and bisulfite modification

DNA from the cell lines KG1, KG1a and MDA-MB-231 and the peripheral blood from a normal volunteer were extracted using the QIAamp DNA blood mini kit (Qiagen, Hilden, Germany), with the added step of digesting the samples for 18 h with 12 μL of 20 mg/mL proteinase K (Worthington Biochemical Corporation, Lakewood, NJ) in 200 μL AL buffer from the DNA extraction kit overnight at 56 °C. Unmethylated control DNA was generated by performing whole genome amplification twice in succession on normal peripheral blood DNA [5, 6]. One nanogram of DNA was amplified using the GenomiPhi DNA V2 amplification kit (GE Healthcare, Little Chalfont, UK) using the manufacturer’s instructions. One microlitre of a 1:10 dilution of this product was then amplified again. CpGenome Universal Methylated DNA (Millipore, Temecula, CA) was used as the fully methylated control. One microgram of DNA was bisulfite modified using the MethylEasy Xceed bisulfite modification kit (Genetic Signatures, North Ryde, Australia) according to the manufacturer’s instructions.

### DNA methylation standards and cell lines

Fully methylated DNA was diluted into unmethylated DNA at 50 and 25% dilutions. KG1 was also included for use with the *CDKN2B* assay and MDA-MB-231 for the *DAPK1* assays as these cell lines are heterogeneously methylated at the respective loci [7, 8]. To ensure accurate mixing of DNA to create a standard series, the concentrations of the fully methylated and unmethylated controls were adjusted after bisulfite modification to amplify at the same Cq in multiple control assays. These assays are in regions devoid of CpG dinucleotides and contain controls for complete conversion, so that they accurately measure bisulfite-modified DNA input. The assays were within *COL2A1* (GTAATGTTAGGAGTATTTTGTGGGTA forward primer and CTACCCCAAAAAAACCCAATCCTA reverse primer) using the same PCR conditions as below used for methylation-sensitive high-resolution melting (MS-HRM) except for annealing at 64 °C and performed for 45 cycles instead of 50 and *HMBS* (GGTTTGATTTTTTGTTTTAGGGTTATT forward primer and TACCACCAATCAACACTCCTCAAA reverse primer) using the same PCR conditions as used for MS-HRM below, except that the annealing and extension were combined at 60 °C for 30 s. Normalisation of standards was performed as described previously [9].

### PCR amplification

All PCRs were performed as a real-time assay with a MS-HRM step on a Rotorgene 6000 (Corbett, Sydney, Australia). PCR was in 0.1 mL tubes with a final reaction volume of 20 μL containing 200 nmol/L of each primer, 200 μmol/L of each dNTP (Fisher Biotec, Perth, Australia), 5 μmol/L SYTO 9 (Molecular Probes), 2.5 mmol/L MgCl_2_, 0.5 U HotStarTaq DNA Polymerase (Qiagen) and 10 ng bisulfite-modified DNA (theoretical amount, assuming no loss of DNA during bisulfite conversion) in the buffer supplied with the polymerase (1×). PCR began with a hold at 95 °C for 15 min followed by 50 cycles of 95 °C for 20 s; 56, 58, 60, 62 or 64 °C for 20 s and 72 °C for 30 s. HRM was followed immediately with a hold at 95 °C for 1 min and 72 °C for 1 min and then an analysis step from 65 to 95 °C rising 0.2 °C/s and a 1 s hold at each increment.

The primer sequences for the *CDKN2B* MS-HRM assay were 5′-TTAGTT**C**GTTTGTAGGGTTTTTATTGGT-3′ and 5′biotin-TAC**G**ACTTAAAACCCC**G**TACAATAACC-3′ [10] corresponding to [GenBank: AL449423] nucleotides 99,845 to 99,958. The primer sequences for the *DAPK1* assay were 5′-GTTAGG**C**GTTTTTTTTTAGAAGTAATTTAGG-3′ and 5′biotin-**G**CC**G**ACCCCAAACCCTACC-3′ [11] corresponding to [GenBank: AL161787] nucleotides 46,924 to 47,105. The bases in bold are the sites at which the primers varied. Primers were obtained from Geneworks (Adelaide, South Australia) except for the abasic and biotinylated primers which were from Sigma-Aldrich. The annealing temperatures tested began at 58 °C and increased in 2 °C increments up to 64 °C, except for the mismatched primers for *CDKN2B*, which began at 54 °C and ended at 60 °C.

### Bisulfite pyrosequencing

The entire PCR product from the MS-HRM was used for bisulfite pyrosequencing. The *DAPK1* sequencing primer was 5′-AGTGTGAGGAGGATAGT-3′, and the *CDKN2B* sequencing primer was 5′-GTTTTTTTTTAGAAGTAATTTAG-3′. The pyrosequencing reaction was performed on a PyroMark Q96 instrument (Qiagen) using the Pyro Gold Reagents PyroMark Q96 (Qiagen). Purification and processing of the biotinylated single-stranded DNA was performed according to the PyroMark Q96 instructions.

## Results

**Fig. 1:**
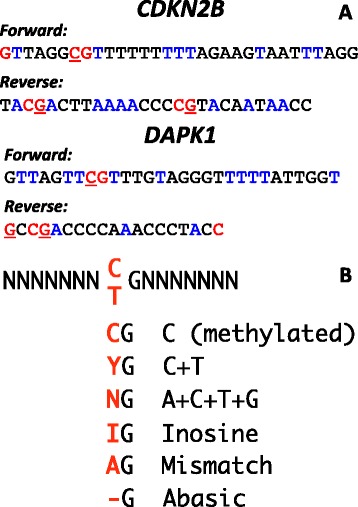
PCR primers for *CDKN2B* and *DAPK1*. **a** CpG dinucleotides are shown in red, and the position matching the cytosine in the dinucleotide is underlined (C in forward primers and G in reverse). Blue thymines (forward primers) and adenines (reverse primers) match cytosines in the DNA sequence that are not part of CpG dinucleotides and therefore undergo bisulfite modification. **b** Each of the six primer types tested are shown. The letters in red indicate the substitution in the forward primer using IUPAC nomenclature. The flanking *N*s denote the flanking sequence. For the mismatch primer (with a mismatched base), the base was arbitrarily chosen as an A in the forward primer (and a T in the reverse primer). In the primer with the abasic site, there is no base attached to the deoxyribose while the regular DNA backbone is intact

We have previously published the sequences of the primers for the methylation-sensitive high-resolution melting (MS-HRM) assays for the two genes analysed here: *DAPK1* and *CDKN2B* [10, 11]. Due to the CpG density and other constraints such as amplicon length, the placing of primers spanning CpG dinucleotides was unavoidable. The positions of the CpG dinucleotides in the sequence bound by the primers had been kept as far from the 3′ end as possible to minimise their effect on any potential bias. Each of the forward primers in both assays overlie a single CpG dinucleotide close to the 5′ end of the primer, while each of the reverse primers overlies two CpG dinucleotides. The *DAPK1* reverse primer has both CpG dinucleotides close to the 5′ end, while the *CDKN2B* reverse primer has a CpG dinucleotide at the 5′ end and a CpG dinucleotide 3′ of the centre (Fig. 1a).

Six different base substitutions at the nucleotide(s) corresponding to the cytosine of the CpG site were used, as listed in Fig. 1b. These included (i) retaining the cytosine (C), the introduction of (ii) a C/T (Y) degeneracy (A/G (R) in the reverse), (iii) an A/T/C/G (N) degeneracy, (iv) inosine (I), (v) a mismatch (A in the forward primer and T in the reverse) and (vi) an abasic site (illustrated in Fig. 1). The abasic site is a modification available from most commercial primer manufacturers and consists of a furan ring at the specified position in the primer sequence (i.e. a sugar with no base attached).

PCR amplification bias may be also manipulated by altering the annealing temperature, as an increase in temperature increases the requirement for an exact match of the primer sequence [2, 12, 13]. For both assays, amplification was possible within annealing temperatures between 58 and 64 °C. However, the mismatched primers for *CDKN2B* could only amplify DNA with annealing temperatures between 54 and 60 °C.

We used bisulfite pyrosequencing to quantify PCR amplification bias [10]. Bisulfite pyrosequencing requires one of the PCR primers to have a biotin label on the 5′ end. Using MS-HRM to compare amplification products with and without biotin labelling, we confirmed our previous results that the biotin labelling has no effect on the amplification bias [10] nor did it change the bias with altered annealing temperatures (data not shown).

To assess PCR amplification bias for mixtures of fully methylated and unmethylated templates (homogeneously methylated DNA), dilutions of fully methylated DNA into unmethylated DNA were used to create a standard series. The dilutions used were 100 (undiluted), 50, 25 and 0%. To ensure these dilutions were representative of real biological samples, the behaviour of the 25% dilution was compared to that of the cell line KG1a for the *DAPK1* assay, as KG1a has a methylation level of 25% (homogeneous pattern) at this locus. The behaviour of the KG1a cell line and the 25% dilution for the *DAPK1* assay was similar for all PCR primers across all PCR conditions (data not shown).

**Fig. 2:**
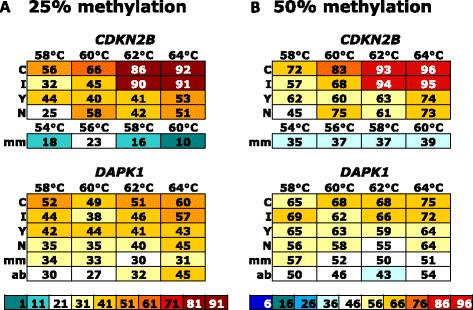
Effect of substitutions and temperature on bias for homogeneously methylated templates. The rows represent the different substitutions at the variable sites. C primers matched the methylated template sequence (C in the forward primer, G in the reverse), I primers had inosines, Y primers had a C/T degeneracy (A/G in the reverse), N primers had totally degenerate bases (A/T/C/G), mm primers had mismatches (A in the forward primer and T in the reverse primer) and ab primers had abasic sites. The columns represent the annealing temperatures tested. For each assay, the average methylation percentage as measured at each CpG by bisulfite pyrosequencing across the amplicon is shown. The final CpG in both assays was not used in the calculations of the average methylation across the amplicon as the pyrosequencing measurement was consistently lower than expected for a homogeneously methylated template and was called as inaccurate by the software. The annealing temperature increases along the *x*-axis by 2 °C for each step as indicated. The mismatched (mm) primers for *CDKN2B* used different annealing temperatures as indicated but were still used in 2 °C increments. The colour scales were set so that the target measurement reflecting the actual level of methylation is shown as white (25 in the 25% methylated templates (panel **a**) and 50 in the 50% methylated templates (panel **b**)). Because of this, the same methylation values have different colours for the 25 and 50% dilutions. The scales at the bottom represent the colour coding for the 25 and 50% mixtures, respectively. The numbering in each scale indicates the lowest value for each colour, i.e. 11 represents 11–20

The approach of using limited cytosines at CpG dinucleotides to match methylated sequences in the forward primers (and guanines in the reverse primers) has been used to compensate for a potential bias towards the amplification of unmethylated DNA when “methylation-independent primers” are used [12, 14, 15]. However, in these experiments for both primer pairs assessed, primers with sequences at the variable position matching the methylated templates overestimated the DNA methylation content (Fig. 2) at all annealing temperatures, with the effect being more pronounced at the higher temperatures.

As the annealing temperature increased, the overestimation of the methylation content increased, until almost only fully methylated sequences were amplified at the highest two annealing temperatures (62 and 64 °C) in the case of *CDKN2B*. For the *DAPK1* assay, the overestimation only reached 25–30% above the actual DNA methylation content. The increase in the estimation of DNA methylation with increasing annealing temperature is more pronounced in the *CDKN2B* assay, presumably because of the more 3′ position of the CpGs in the reverse primer.

Primers with inosines at the potentially methylated sites performed almost identically to the primers with cytosines, i.e. they also overestimated the degree of methylation, and this effect increased with increasing annealing temperature (Fig. 2).

The most commonly used solution in the literature when CpG dinucleotides within primers are unavoidable is to use a degenerate C/T (Y) in the forward primer and A/G (R) in the reverse primer [4]. We found that these primers also overestimated DNA methylation, although not to the same extent as the cytosine- and inosine-containing primers. For the *CDKN2B* assay, there was a mild increase in the methylation estimate with increasing annealing temperature; however, with the *DAPK1* assay, the DNA methylation measurement was independent of the annealing temperature (Fig. 2).

An up-to-now untested variation to using a degenerate C/T is to use totally degenerate bases (N) at CpG cytosine (guanine in the reverse primer) positions. At the lowest annealing temperature (58 °C), there was no major bias in either direction. As the annealing temperature increased, the degree of overestimation of DNA methylation increased, especially for *CDKN2B* (Fig. 2). This increase was similar but less than that seen with the Y-degenerate primers. Thus, although these primers have an inherent bias towards methylated templates, it can be minimised with lower annealing temperatures (Fig. 2).

The primer sets that contained mismatches at the variable sites had a poor amplification efficiency. The addition of an extra five cycles to the PCR protocol was required to generate enough PCR product to enable bisulfite pyrosequencing. For *DAPK1*, there was minimal variation in results across the range of annealing temperatures and the accuracy was close, with a minor overestimation for the 25% control that is almost within the error range for bisulfite pyrosequencing. However, DNA methylation was underestimated across all conditions for *CDKN2B* (Fig. 2), the only instance of underestimation for any homogeneous DNA methylation. The *CDKN2B* assay also required a 4 °C drop across the series of annealing temperatures for successful amplification.

The *CDKN2B* primers with abasic sites at the variable sites failed to amplify any product at all, so the forward and reverse primers were tested individually with the corresponding cytosine (forward)- or guanosine (reverse)-containing primer as the other primer, in order to determine which primer was responsible for the failure. The forward primer with the abasic site successfully amplified the DNA with the guanosine-containing reverse primer. The reverse primer with abasic sites failed with the C-containing forward primer, indicating that the central abasic site is close enough to the 3′ end of the primer to interfere with PCR. No further testing was done for these *CDKN2B* primers.

Amplification at *DAPK1* for the primers with abasic sites remained largely annealing temperature independent within the error range of bisulfite pyrosequencing. The 50% methylation standard measurements were accurate at all temperatures. At the highest annealing temperature, however, there was significant overestimation of DNA methylation for the 25% methylated standard. This result was reproducible for all replicates performed.

### Heterogeneously methylated target regions

Unlike the case for homogeneous methylation, meaningful standards for heterogeneous methylation are not feasible as the patterns of epialleles vary considerably between samples [10, 11]. In this study, we used cell lines that were heterogeneously methylated for the regions in question as a model system. The primers and conditions that were found to be least biased for the amplification of homogeneously methylated DNA were assumed to be the least biased for the amplification of heterogeneously methylated DNA. These conditions were chosen as a reference point, and all other primers and conditions were compared to the results for these conditions. Strikingly, heterogeneously methylated samples were less sensitive to the changed PCR conditions compared to fully methylated DNA in a background of unmethylated DNA, with all primer substitutions.

For *CDKN2B*, the KG1 cell line, which shows heterogeneous methylation at this locus [7], was used. The results were normalised relative to the N-degenerate primers at the 58 °C annealing temperature. Regardless of the primers or conditions used, there was minimal, if any, bias detected at all, except for the cytosine- and inosine-containing primers. The cytosine-containing primers overestimated the methylation at each variable position by 10–30% at the higher annealing temperatures. The inosine-containing primers gave similar results. As with the homogeneous methylation experiments, this is likely to be due to the central CpG in the *CDKN2B* reverse primer, which may be increasing the bias towards methylated templates.

The Y-degenerate primers were largely independent of temperature, but they marginally overestimated the methylation at the highest annealing temperature and marginally underestimated the methylation at the lowest annealing temperature. The N-degenerate primers that were used for the standard were also largely independent of temperature but also marginally overestimated the methylation at the highest annealing temperature. No real bias at any temperature was seen using the mismatch-containing primers, but due to the poor amplification, these would not normally be used.

For *DAPK1*, the MDA-MB-231 cell line, which shows heterogeneous methylation at this locus [8], was used. The *DAPK1* results for MDA-MB-231 were similar to only minor differences between the conditions. Results were again normalised relative to the N-degenerate primers at an annealing temperature of 58 °C. Regardless of the primers or conditions used, there was again minimal, if any, bias detected at all, except for the inosine- and abasic-containing primers that surprisingly underestimated the methylation at some of the variable sites at the highest annealing temperatures. Unlike *CDKN2B*, pronounced bias towards methylation with cytosine and inosine at 62 and 64 °C was not seen for *DAPK1*. Overall, the Y-degenerate primers gave the most stable result across the annealing temperatures.

## Discussion

While the problem of PCR amplification bias in the analysis of DNA methylation has long been recognised (reviewed in [16]), it has been overlooked in most studies. The problem is often acute in the study of CpG-rich regions, where the usual advice of avoiding CpGs in primer sequences is often not implementable, particularly if targeted regions need to be amplified for the analysis of clinical samples. In many cases, the starting material is already highly fragmented before bisulfite conversion (such as the DNA extracted from plasma or FFPE tissues), limiting the optimal amplicon length and thus further limiting the options for primer placement.

In this report, we examined the effect of different base substitutions at the cytosine position of CpGs under the primer on the representational amplification of methylated templates. We studied the promoter regions of two genes that play important tumour suppressor roles in cancer, *CDKN2B* and *DAPK1* [17–19]. These genes are often heterogeneously methylated [11, 20–23] providing an ideal scenario to comprehensively examine the primer design and temperature parameters influencing amplification bias both in the heterogeneously methylated context and in the homogeneously methylated context.

We have previously published primers for the two promoter regions in this study [10, 11]. Due to the CpG density and other constraints, the placing of primers over CpG dinucleotides was unavoidable. We now used six different substitutions at the potentially methylated cytosine sites overlaid by these primers in order to identify those that might be least biased.

When we looked at homogeneously methylated regions, we saw major influences of both the primer type and the annealing temperature. In addition, the actual number and placement of CpGs in the primers had a major impact on the bias.

In the first set of primers tested, the variable sites matched methylated templates (C in the forward and G in the reverse primer). This approach of using limited CpGs in the primers may be used to compensate for a potential natural bias towards amplification of unmethylated DNA for the design of methylation-independent primers for unbiased amplification [12, 14, 15]. Our results clearly show that these primers are biased towards the amplification of methylated sequences, particularly at higher temperatures.

Inosines at the variable sites were included in the second set of primers as they have been used in the literature as a substitute for degenerate bases [24], including the analysis of bisulfite-modified DNA [25]. The expectation is that inosine should pair with both cytosine and thymine equally. However, inosines preferentially bind to cytosine, thymine and adenine over guanine. The primers containing inosines were not as biased as the primers containing cytosines but still overestimated methylation, resulting in estimates 20–30% higher than the actual level of methylation at the lower temperatures. They behaved like the primers with cytosine at the variable site, when tested at the higher temperatures.

**Fig. 3:**
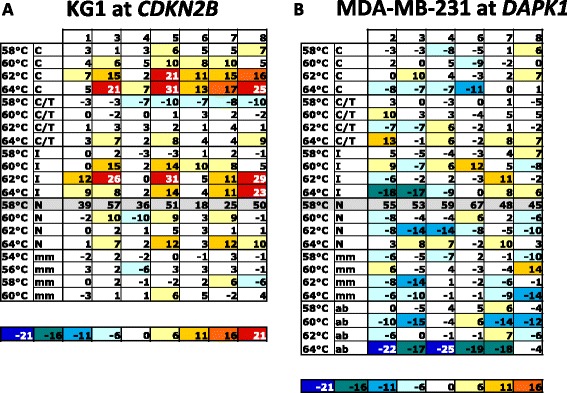
Effect of substitutions and temperature on bias for heterogeneously methylated templates. Two cell lines (KG1 and MDA-MB-231) were chosen that were respectively heterogeneously methylated for *CDKN2B* (panel **a**) and *DAPK1* (panel **b**). The rows represent the different substitutions at the variable sites across the range of annealing temperatures used for each substitution. The numbers at the top are the CpG dinucleotides ordered across each amplicon. Only the CpGs that are not always methylated in the cell lines are shown. The final CpG in both assays has been omitted as the pyrosequencing measurement was called as inaccurate by the software. The annealing temperature increases along the *y*-axis by 2 °C for each step as indicated. Based on the homogeneous methylation data, where the lowest annealing temperature for the N-containing primers was the most accurate measurement of DNA methylation, the same conditions have been chosen as the standard for the measurement of DNA methylation in these tables (shaded in grey with the actual values measured). The values shown in the rest of the figure are deviations from the standard set of measurements (the raw data is shown in Additional file 1: Figure S1). The scale has been set so that minimal deviations from the standard (±5%) are in white. The colour scale indicating deviation from the standard is common for the two tables

The most common approach to overcome the bias problem in the literature is to include a C/T (Y) degeneracy in the forward primer (A/G (R) degeneracy in the reverse primer) at the variable site [4]. Our results clearly show that this approach does not lead to unbiased amplification of DNA (Fig. 3), consistent with the previously reported results of Shen et al. [13]. The primers containing a Y-degeneracy were still biased, but less biased than the primers containing cytosines, at each temperature point.

The fourth set of primers was totally degenerate (N) at the variable sites. The N-degenerate primers gave similar results to the Y-degenerate primers. However, at the lowest annealing temperature tested, the N-degenerate primers were the most accurate for the *CDKN2B* assay. The fifth set of primers had abasic sites at the variable sites. For the *DAPK1* assay, the primers containing abasic sites outperformed the N-degenerate primers. In fact, the *DAPK1* primers containing abasic sites did not display any bias for almost all conditions for both the 50 and 25% methylated standards. However, the N-degenerate primers may be more attractive for use as unbiased primers (when used at low annealing temperatures) due to there being fewer constraints relative to the abasic site-containing primers.

The last set of primers was mismatched at the variable sites. The *CDKN2B* assay resulted in a consistent underestimation of DNA methylation content across all conditions used. The *DAPK1* assay, however, resulted in a small overestimation of methylation, and as with the *CDKN2B* assay, this was consistent over all conditions tested. However, as amplification was inefficient with these primers and PCR optimisation was difficult, they would not be a usual recommendation.

Surprisingly, while the primers containing abasic sites appear to deliver the most accurate results and are the most resistant to changes in PCR conditions, there is only one report of their use in the literature [26]. This may in part be due to the fact that the use of Y- and R-degeneracies appears to be the most logical and the most commonly used is the use of Y- and R-degeneracies. The ability of oligonucleotide manufacturers to synthesise primers with abasic sites makes this an option, albeit an expensive one. The absence of a temperature effect reinforces this observation. While the use of abasic site-containing primers seems to be the best option, it does have some drawbacks. They will not prime reactions in some sequence contexts as has been demonstrated with the *CDKN2B* assay, which is suggestive of the DNA polymerase having difficulties extending from abasic sites.

Whenever a bias was evident, it nearly always resulted in overestimation of methylation except for the mismatched primers, which suffered from poor amplification. The primers that resulted in the highest overestimations were the primers fully complementary to methylated templates (i.e. containing cytosines). As the primers match the sequence of the methylated but not the unmethylated templates, they would preferentially bind to methylated targets. When assessing PCR bias in the *CDKN2B* and the *DAPK1* assays, bias towards methylated sequences in the *CDKN2B* assay was more prevalent. The *CDKN2B* assay was more biased towards methylated sequences than the *DAPK1* assay, most likely due to the extra CpG in the middle of the *CDKN2B* reverse primer, enhancing selectivity towards methylation.

Whereas we have only investigated two gene loci, the relationship between both the number of CpGs and their position in the primers and the severity of bias provides a good indicator of the potential bias in other assays. It would not be expected that the direction of bias (i.e. over- or underestimation of methylation) should change with different primers, as it is likely that it is the inclusion of a CpG dinucleotide in the primer sequence regardless of position would be causing bias towards methylation at higher temperatures.

Furthermore, although non-CpG cytosine methylation [27–29] was not considered here, we see no reason why the same design strategies (applied to both strands) would not hold, as the effect should be solely related to the cytosine base.

A key finding was that the lowest temperature gave on the whole the least biased results. Increasing the annealing temperature affects the assay stringency and therefore the PCR bias. For both the *CDKN2B* and the *DAPK1* assays, the cytosine-, Y-degenerate, N-degenerate and inosine-containing primers are prone to this, with increasing stringency leading to increased overestimation of methylation. This suggests that these primers are inherently biased towards amplification of methylated templates, regardless of the conditions used. In some cases, this bias towards methylation with increasing temperature can be exploited to assist the detection of low-level methylation.

Shen et al. [13] also demonstrated a similar phenomenon in the primer sets that they assessed. They examined primer pairs without CpG dinucleotides, those that had a Y-degeneracy and combinations of both. Interestingly, the temperature sensitive bias was observed in all three types of primer combinations, i.e. bias towards methylated sequences increased with temperature. This indicates that even primers that do not contain CpGs have some inherent bias towards methylated templates with increasing temperature.

Interestingly, heterogeneously methylated loci seem to be less sensitive both to the choice of primer substitution and to the variation in annealing temperature. The heterogeneously methylated samples may be less “susceptible” to effects introduced by primers as the binding of primers in this situation would be more random than in the homogeneous methylation/non-methylation context. This would be particularly apparent when primers with at least two CpGs are used.

## Conclusions

We examined the effects on PCR bias of a range of substitution strategies at potentially methylated cytosine sites in PCR primers together with the examination of different annealing temperatures. Amplification from both homogeneously methylated and heterogeneously methylated templates was examined.

Homogeneous methylation patterns were shown to be highly susceptible to biased PCR amplification, confirming the observations reported by others for some of these substitutions [13]. This is important for assessing methylation levels especially for imprinting disorders where deviations from equivalency may be clinically important [30]. Using abasic sites in primers might in many cases be the preferred solution, as amplification remains unbiased over a range of annealing temperatures. However, there will be circumstances where the position of CpGs in the primer will not allow the use of abasic sites as seen in the *CDKN2B* assay. In these instances, designing primers with an A/C/T/G (N) degeneracy at the variable sites followed by optimisation of the PCR assay to use the lowest possible annealing temperature appears to be the best alternative.

Importantly for much of the DNA methylation data in the cancer literature, heterogeneous methylation, which is more common than homogeneous methylation [23, 31, 32], is comparatively resistant to PCR bias introduced by the primers and annealing temperatures. Nevertheless, the lowest annealing temperature gave the least biased results overall across the range of substitutions tested.

These results are applicable to all methods needing an accurate representation of methylation including bisulfite clonal sequencing following PCR. These considerations are also pertinent for multiplexed amplicon approaches as used in massively parallel sequencing.

## Additional file

Additional file 1: Figure S1. The raw heterogeneous methylation data, from which Fig. 3 is derived. This includes full pyrosequencing data for all CpG sites. The colour scheme relates to the raw data and not the deviations from unbiased amplification as shown in Fig. 3. (PDF 45 kb)
