# Supplementary material for: Assessing alternative base substitutions at primer CpG sites to optimise unbiased PCR amplification of methylated sequences
Source: Clin Epigenetics. 2017 Apr 4;9:31. doi: 10.1186/s13148-017-0328-4 (PMC5379501; doi:10.1186/s13148-017-0328-4)
Supplement: Additional file 1: Figure S1. — The raw heterogeneous methylation data, from which Fig. 3 is derived. This includes full pyrosequencing data for all CpG sites. The colour scheme relates to the raw data and not the deviations from unbiased amplification as shown in Fig. 3. (PDF 45 kb) [file 13148_2017_328_MOESM1_ESM.pdf]

A *CDKN2B* in KG1

		1	2	3	4	5	6	7	8	9
58°C	C	42	93	58	39	57	23	30	57	26
60°C	C	43	93	63	41	61	26	35	55	25
62°C	C	46	93	72	38	72	29	40	66	30
64°C	C	44	96	78	43	82	31	42	75	35
58°C	C/T	36	90	54	29	41	11	17	40	10
60°C	C/T	39	93	55	36	52	21	27	48	18
62°C	C/T	40	91	60	39	53	19	29	51	18
64°C	C/T	42	92	64	38	59	22	29	59	23
58°C	N	39	94	57	36	51	18	25	50	22
60°C	N	37	93	67	26	60	21	34	49	22
62°C	N	39	93	59	37	56	21	26	51	22
64°C	N	40	90	64	38	63	21	37	60	31
58°C	ino	39	91	59	33	48	19	27	49	17
60°C	ino	39	93	72	38	65	28	33	55	23
62°C	ino	51	95	83	36	82	23	36	79	30
64°C	ino	48	96	65	38	65	22	36	73	33
54°C	mm	37	93	59	34	51	17	28	49	23
56°C	mm	42	91	59	30	54	21	28	49	21
58°C	mm	39	94	59	35	49	20	31	44	23
60°C	mm	36	92	58	37	57	23	23	54	30

0	10	20	30	40	50	60	70	80	90	100

B *DAPK1* in MDA-MB-231

		1	2	3	4	5	6	7	8	9
58°C	C	88	52	50	51	76	62	49	51	80
60°C	C	94	57	53	64	70	58	46	45	72
62°C	C	90	55	63	63	84	64	50	52	77
64°C	C	89	47	46	52	88	56	48	46	73
58°C	C/T	96	58	53	56	83	67	49	40	80
60°C	C/T	93	65	56	62	80	63	53	50	71
62°C	C/T	86	48	46	65	73	65	49	43	75
64°C	C/T	89	68	52	65	85	65	56	52	71
58°C	N	85	55	53	59	75	67	48	45	78
60°C	N	85	47	49	55	71	73	50	39	75
62°C	N	84	47	39	45	64	59	43	35	64
64°C	N	89	58	61	66	90	65	58	48	69
58°C	ino	91	60	48	55	71	64	52	52	74
60°C	ino	88	64	46	65	75	79	53	37	76
62°C	ino	93	49	51	61	69	64	59	43	78
64°C	ino	78	37	36	50	69	67	56	51	75
58°C	mm	89	49	48	52	79	69	47	35	70
60°C	mm	92	61	48	63	85	64	44	59	60
62°C	mm	89	47	39	60	74	65	42	44	70
64°C	mm	93	49	43	58	80	66	39	31	75
58°C	sp	86	55	48	63	84	72	54	41	68
60°C	sp	92	45	38	55	66	73	34	33	66
62°C	sp	87	49	53	58	70	66	55	39	81
64°C	sp	80	33	36	34	53	48	30	41	74

0	10	20	30	40	50	60	70	80	90	100
